# Effects of COVID-19 lockdown on low back pain intensity in chronic low back pain patients: results of the multicenter CONFI-LOMB study

**DOI:** 10.1007/s00586-021-07007-8

**Published:** 2021-10-04

**Authors:** Florian Bailly, Stéphane Genevay, Violaine Foltz, Amélie Bohm-Sigrand, Alain Zagala, Julien Nizard, Audrey Petit

**Affiliations:** 1 Pain Unit, Rheumatology Department, Pitié Salpêtrière Hospital, AP-HP, 47-83 boulevard de l’hôpital, 75013 Paris, France; 2 Division of Rheumatology, Beau Sejour Hospital, Geneva University Hospitals, 14, CH-1205 Geneva, Switzerland; 3 Rheumatology Department, Pitié Salpêtrière Hospital, AP-HP, 47-83 boulevard de l’hôpital, 75013 Paris, France; 4 Rheumatology Department, Dijon University Hospital, 14 rue Gaffarel, 21000 Dijon, France; 5 Rheumatology Practice, 17 Boulevard Agutte Sembat, 38000 Grenoble, France; 6 Pain, Palliative and Supportive Care Department, University Hospital of Nantes, 1 Place Alexis-Ricordeau, 44093 Nantes, France; 7 University of Angers, Inserm, EHESP, Irset, UMR-S 1085, Angers, France; 8 Department of Occupational Medecine, University Hospital of Angers, 4 rue Larrey, 49933 Angers cedex 9, France

**Keywords:** COVID-19, Low back pain, Physical activity, Lockdown experience

## Abstract

**Purpose:**

The COVID-19 pandemic and the extended lockdown are associated with numerous changes in behavior and lifestyles. The objective was to assess the impact of the first lockdown on LBP course among chronic LBP patients.

**Methods:**

Descriptive and analytical, cross-sectional, multicenter study, conducted by questionnaire from mid-May to end of June 2020 among patients treated for chronic LBP in 6 French and 1 Swiss center. Collected data concerned changes in LBP intensity during lockdown, lockdown experience, physical activity (PA) practice and sedentary lifestyle prior and during lockdown, recourse to care, consumption of psychoactive substances for LBP, and professional activity and its conditions during lockdown.

**Results:**

360 participants (58.6% women, 52.1 ± 13.4 years) were included of which 65% were active (63% keep on working of which 54% teleworked). LBP got worse in 41.1%, mean VAS went from 49.5 ± 21.6 before to 53.5 ± 22.4 during lockdown (*p* < 0.001) and needed increase of treatment by 29% but very few people increased their consumption psychoactive substances for analgesia. Half of participants had well-experienced lockdown. Findings revealed a significant decrease in PA and increase of sedentary during lockdown (*p* < 0.0001). Good experience of lockdown was associated with LBP improvement (OR = 0.6 [0.3–0.9]) and decrease of PA with LBP worsening (OR = 1.9 [1.1–3.2]). Teleworking was also associated with LBP worsening. Gender, age, or BMI did not influence LBP course.

**Conclusion:**

These findings indicate that chronic LBP people suffered from increase in self-perceived LBP during lockdown and help to better understand the factors associated with their condition.

**Supplementary Information:**

The online version contains supplementary material available at 10.1007/s00586-021-07007-8.

## Introduction

The COVID-19, which was declared a global pandemic on 11th March 2020 by the WHO has quickly become a serious challenge, affecting all societies. The context of COVID-19 pandemic generates health concerns but is also associated with numerous changes in behavior and lifestyles, in particular, due to the extended lockdown [1–5]. Although social lockdown remains the best non-pharmacological solution to decelerate the fast transmission of the virus, it is likely generating negative consequences on mental health and lifestyle behaviors. Thus, recent studies demonstrated that lockdown could dramatically impact lifestyle activities globally, including participation in sports and physical activity (PA) practice [1, 6–8].

Numerous studies have shown that the onset of low back pain (LBP), its recurrence, and, even more, its evolution towards chronicity, are the consequences of a complex interrelation between physical, psychological, social, and professional factors, known as “bio-psycho-social model” [9]. If the prevention of LBP and chronic LBP, as well as their management, is based primarily on the practice of regular sports and PA, other psychological (stress, anxiety, depression, catastrophizing), socioeconomic factors (low level of education or resources), professional (ergonomic factors, job dissatisfaction), etc. also have a major role in the LBP course [10, 11]. Though not identical conditions or contexts, we can cautiously suppose that COVID-19 pandemic, and more particularly the lockdown that we underwent in spring 2020, had and will have significant consequences for people with chronic LBP as that have been suggested in previous studies on chronic pain patients [8, 12]. However, the interference of the different factors related to lockdown had potentially different consequences at the individual level. It seemed therefore difficult to predict what would be the evolution of lumbar symptoms in this population. Indeed, if we can speculate consider that the decrease in PA practice and a bad lockdown experience in this context of insecurity could lead to a pain increase, we could just as well speculate consider that the decrease in stress at work, heavy physical work, or even commuting times, on the contrary, could have a positive effect.

The main objective of this study was to assess the impact of the spring 2020 lockdown on LBP intensity course in chronic LBP patients. The secondary goal was to study the impact on LBP course of the pandemic experience, behavior and lifestyles, and occupational conditions during lockdown.

## Methods

### Study design and setting

We report findings on a descriptive and analytical, cross-sectional, multicenter study on the impact of lockdown on the LBP intensity in chronic LBP patients (CONFI-LOMB study). CONFI-LOMB was conducted by questionnaire, from May 12 to June 30, 2020. The project’s steering group was composed of seven French and Swiss rheumatologists, all members of the French Rheumatology Society. Seven centers were involved in the study: University Hospital of Angers, Dijon, Genève, Nantes, two of Paris as well as private rheumatology consulting practice in Grenoble. Lockdown time and the limitation were similar in both countries.

### Participants screening and inclusion

Inclusion criteria were: (1) to be an adult (men or women), (2) to have undergone a consultation for a common chronic LBP between January 1, 2020, and March 17, 2020 (start of the French lockdown) included. Common chronic LBP was defined as non-specific LBP (i.e. LBP for which the specialist has ruled out malignant, traumatic, infectious, or inflammatory origin thanks to clinical, biological, and/or imaging explorations) lasting for at least 3 months without improvement, (3) consent to participate in this research.

Exclusion criteria were: to suffer from another chronic pain syndrome interfering with daily life activities (fibromyalgia, etc.), to have comorbidities limiting the practice of sports and PA (severe heart or respiratory diseases, claudication due to lower limbs arteriopathy, etc.), poor understanding of the French language and inability to answer a questionnaire (illiteracy, no internet or phone access, etc.).

The physicians contacted eligible patients by phone or sent them a form or an electronic version of the questionnaire after sending an information letter. When done by phone, the physicians provided oral information on the study and answered questions. The first part of the questionnaire underlines the goal of the study and the contact details of the person to contact in case of questions.

### Ethical aspects

During the informed consent process, survey participants were assured all data would be used only for research purposes. Participants’ answers were anonymous and confidential. Additionally, participants were able to stop study participation at any stage of the questionnaire. If doing so, their responses would not be registered. Participants were requested to be honest in their responses.

This research involving the human being was qualified as non-interventional research because it was solely based on the completion of a questionnaire. The study file was submitted to the Ile-de France X Committee for the Protection of Persons who issued a favorable opinion to implement this research on May 11, 2020 (identification N° 2020-AO1417-32). ClinicalTrials.gov identifier: NCT04406363.

### Outcomes and risk factors measurement

An ad hoc questionnaire was prepared in accordance with the available literature and the feedback from patients collected by the members of the study's steering group during lockdown. The questionnaire was composed of 32 questions divided into seven parts: (1) sociodemographic data, (2) condition and experience of lockdown, (3) medical status vis-à-vis of the coronavirus, (4) anthropometric and LBP pain data, (5) consumption of care and psychoactive substances, (6) time spend for PA and sedentary activities, in accordance with usual measurement scales, (8) occupational condition and consequences felt on LBP. The questionnaire was completed in French from May 12 to June 30, 2020.

The primary outcome was a change in LBP intensity prior to the lockdown and during it assessed by a 7-point Likert scale (from much improved to much worsened). A clear definition and a model of response were done to help the subjects answer this question. The second outcomes were: LBP intensity before and during lockdown (Visual Analog Scale (VAS)), the experience of lockdown, the PA practice (sports and leisure) before and during lockdown (less than 2 h a week, from 2 to 4 h a week, more than 4 h a week), time spent in sedentary activities (less than 3 h a day, from 3 to 7 h a day, more than 7 h a day), occurrence of a SARS-CoV-2 infection and its level of severity, recourse to care, consumption of medication and psychoactive substances intended for LBP analgesia, continuation of occupation and its conditions (at the workplace, teleworking).

### Analyses

Given the exploratory nature of this study, and due to the lack of data at the time of the study setting, the number of subjects required to ensure representativeness was estimated, empirically with regard to the active lines of the participating centers, at 50 patients per physician (350 subjects in total).

Results were expressed as mean ± standard deviation for continuous data or as count and percentage for categorical data. A paired t-test was employed to compare VAS of patients before and during lockdown. Wald Chi-square tests were used for comparing categorical variables. Due to the low number of people within some categories of evolution of LBP, the modalities “much improved”, “moderately improved”, “slightly improved”, and “stable” were combined within the same “stable or improved pain” modality, and the modalities “slightly worsened”, “moderately worsened” and “much worsened” were combined within a second “worsened” modality. The association between risk factors and LBP course (worsening *versus* unchanged or improvement) was estimated using logistic regression. Significant variables with a *p*-value under 0.20 in univariate analysis were included in the multivariate logistic regression model. Age, sex, and body mass index (BMI) were forced in the final model. The significant level (*p*) in the final model has been defined as 0.05. Data analyses were performed with version 3.6.1 of the R software.

## Results

### Sample description

Among patients who met the inclusion criteria, a total of 360 were offered the opportunity and accepted to participate in this study. The lockdown duration was the same for every participant. The sample consisted of 149 men and 211 women, from rural and urban areas of the concerned regions, with a mean age of 52.1 ± 13.4 years (from 19 to 91 years). BMI was comprised between 14.7 and 44.4 kg/m^2^ with a mean of 26.1 ± 4.9 kg/m^2^ and remained stable during lockdown. The large majority of the participants had suffered from LBP for more than two years. Most participants lived in couple (71.8%) and had an occupation (65.0%). Among the 230 workers, 63% keep on working of which 54% teleworked during lockdown. Near 58% of teleworkers declared to have a dedicated homework station and 47% to have suitable equipment for teleworking. Half of the participants (51.7%) declared having well-experienced lockdown. Finally, 6.4% of people were infected by the SARS-CoV-2 with a low rate of severe symptoms (Table 1).

**Table 1:** Patients characteristics

	*N*	%*
Gender		
Male	149	41.4
Female	211	58.6
Age		
18–34 years	33	9.2
35–49 years	134	37.2
50–64 years	125	34.7
≥ 65 years	68	18.9
BMI before lockdown		
< 25 kg/m^2^	156	43.6
≥ 25 kg/m^2^	202	56.4
LBP duration		
3–6 months	7	2.0
6–24 months	50	14.1
> 24 months	297	83.9
Marital status		
Couple	255	71.8
Single	100	28.2
Occupational status		
Active	230	65.0
Non-active	124	35.0
Keep on working during lockdown		
Yes	129	63.2
No	75	36.8
Telework		
Yes	67	54.0
No	57	46.0
Workstation dedicated for telework		
Yes	39	58.2
No	28	41.8
Equipment adapted to telework		
Yes	28	46.7
No	32	53.3
Lockdown experience		
Good	184	51.7
Neither good or bad	104	29.2
Bad	68	19.1
SARS-COV-2 infection		
Yes	23	6.4
No	335	93.6
Severity of SARS-COV-2 infection		
Few or no symptoms	11	47.8
Discomfort in ADL	7	30.4
Hospitalization	1	4.3
Intensive care	0	0

*ADL* Activities of daily living; *BMI* Body mass index; *LBP* Low back pain

*Percentages without missing data

### LBP, recourse to care and treatment, and physical activity during lockdown

LBP was unchanged or improved in almost 59% while more than 41% of them got worse. The mean pain VAS course went from 49.5 ± 21.6 before lockdown to 53.5 ± 22.4 during lockdown (p < 0.001). Near 30% of participants visited a doctor or increased their treatment for LBP while 12.1% decreased it during lockdown. Very few patients increased their consumption of alcohol, tobacco, cannabis, or anxiolytics to decrease pain (Table 2). Lockdown significantly impacted participation in PA practice which globally decreased during lockdown (*p* < 0.0001). Overall, 30% of patients decreased their practice of PA while less than 14% increased it. In parallel, sedentary lifestyle significantly increased during lockdown (*p* < 0.0001) with a proportion of people spending more than 7 h a day in sedentary activities going from 19 to 32% (Table 3).

**Table 2:** LBP and repercussions during lockdown

	*N*	%
Course of LBP		
Much improved	11	3.1
Moderately improved	22	6.1
Slightly improved	18	5.0
Stable	161	44.7
Slightly worsened	65	18.1
Moderately worsened	50	13.9
Much worsened	33	9.2
VAS (/100)		
Before lockdown (mean ± sd)	49.6 ± 21.5	< 0.001^a^
During lockdown (mean ± sd)	53.5 ± 22.4	
Increase of treatment /consultation for LBP		
Yes	103	29.0
No	252	71.0
Decrease of treatment for LBP		
Yes	41	12.1
No	297	87.9
Increase of Tabaco for LBP		
Yes	24	7.2
No	311	92.8
Increase of alcohol for LBP		
Yes	18	5.4
No	316	94.6
Increase of cannabis for LBP		
Yes	2	0.6
No	328	99.4
Increase of anxiolytics for LBP		
Yes	52	15.1
No	292	84.9

*LBP* Low back pain; *VAS* Visual Analog Scale

^a^Paired *t*-test

**Table 3:** Physical activity and sedentary lifestyle

	Before lockdown		During lockdown		*P* value^a^
	N	%	N	%	
Physical activity					
< 2 h/week	121	34.4	170	49.0	< 0.0001
2–4 h/week	118	33.5	84	24.2	
> 4 h/week	113	32.1	93	26.8	
Sedentary lifestyle					
< 3 h/day	167	47.9	84	27.7	< 0.0001
3–7 h/day	116	33.2	123	40.6	
> 7 h/day	66	18.9	96	31.7	

^a^Pearson chi-square tests

### Factors associated with LBP course

Univariate analyses show that LBP increased significantly more often in case of bad experience of lockdown, decrease or low practice of PA, and teleworking during lockdown (*p* < 0.20). On the other hand, LBP course was not influenced by sedentary lifestyle or SARS-COV-2 infection (Table 4).

**Table 4:** Related factors with LBP course

	Decrease or unchanged of LBP		Increase of LBP		*p*-value^a^
	*N*	%	*N*	%	
Lockdown experience					
Good	124	59.3	60	40.8	0.0009
Neither good or bad	56	26.8	48	32.7	
Bad	29	13.9	39	26.5	
Physical activity during lockdown					
< 2 h/week	87	42.4	83	58.5	0.011
2–4 h/week	54	26.3	30	21.1	
> 4 h/week	64	31.2	29	20.4	
Evolution of physical activity before/during lockdown					
Increase	33	16.4	14	9.9	0.001
No change	122	60.7	70	49.3	
Decrease	46	22.9	58	40.8	
Sedentary lifestyle during lockdown					
< 3 h/day	55	29.9	29	24.4	0.529
3–7 h/day	71	38.6	52	43.7	
> 7 h/day	58	31.5	38	31.9	
SARS-COV-2 infection					
Yes	12	5.7	11	7.5	0.498
No	199	94.3	136	92.5	
Keep on working during lockdown					
Yes	84	65.6	45	59.2	0.359
No	44	34.4	31	40.8	
Telework					
Yes	39	48.1	28	65.1	0.069
No	42	51.9	15	34.9	
Workstation dedicated to telework					
Yes	25	64.1	14	50.0	0.249
No	14	35.9	14	50.0	
Equipment adapted to telework					
Yes	19	51.4	9	39.1	0.355
No	18	48.6	14	60.9	

^a^Wald Chi-square test

Multivariate analyses highlight that good experience of lockdown (OR = 0.6 CI_95%_ [0.3–0.9]) was a protective factor of LBP worsening and decrease of PA practice during lockdown (OR = 1.9 CI_95%_ [1.1–3.2]) was significantly associated with LBP worsening. On the other hand, gender, age, BMI did not influenced LBP course during lockdown (Table 5).

**Table 5:** Multivariate analysis of low back pain course (worsening *versus* unchanged or improvement) according to patients characteristics and life conditions during lockdown

	Adjusted OR	IC95%	*p*-value^a^
Gender			
Male	1	–	0.255
Female	1.33	0.82–2.16	
Age			
18–34 years	1	–	0.894
35–49 years	1.20	0.53–2.78	
50–64 years	0.98	0.43–2.29	
≥ 65 years	1.17	0.43–3.25	
BMI before lockdown			
< 25 kg/m^2^	1	–	0.515
≥ 25 kg/m^2^	1.17	0.73–1.89	
Course of physical activity practice during *versus* before lockdown			
Increase	0.78	0.37–1.58	0.024
Constant	1	–	
Decrease	1.88	1.12–3.17	
Lockdown experience			
Good	0.55	0.32–0.93	0.004
Neither good or bad	1	–	
Bad	1.45	0.74–2.84	

*BMI* Body mass index

^a^Wald Chi-square test

## Discussion

We set out to understand how the COVID-19 pandemic and associated lockdown restrictions, impacted individuals with chronic LBP in terms of their pain experience. The findings reveal that LBP has remained unchanged or has worsened for the majority of patients during lockdown. Bad experience of lockdown, low or decrease in levels of PA appeared the main associated factors for LBP worsening. Teleworking was also associated with LBP worsening. However, very few people increase their consumption of tobacco or psychoactive substances.

Concerning LBP course, our sample of chronic LBP patients was more adversely affect by lockdown than the general population among which the majority stated that their lower back problems were unchanged (62.6%), a quarter felt better, while 11.9% felt worse in a French Public Health Institute survey (*n* = 1,111) [13]. An Italian national study reported that almost one in two Italians declared having suffered from back pain more often during lockdown [14]. Our findings are also in accordance with those of Fallon et al. who observed that people with chronic pain reported self-perceived increases in levels of pain severity during lockdown in the UK [8]. Regarding mean VAS evolution during lockdown, we assumed that even statistically significant, the course from 49.5 ± 21.6 before, to 53.4 ± 22.4 during lockdown could be interpreted as at the limit of clinical significance and below the minimal clinically important changes in chronic pain intensity measured on a numerical rating scale [15]. Even if VAS is describe as the most sensitive method for measuring pain and can be also used reliably in chronic clinical pain, it should be taken into account that non-specific chronic LBP is especially characterized by undulant course of pain intensity [16]. Our findings could therefore only be related to the usual course of VAS in chronic LBP patients.

Previous studies have found a significant effect of the COVID-19 pandemic on social participation and life satisfaction scores [1–5] and the decrease in individual well-being has already been observed during similar pandemic crises (2002–2004 SARS outbreak) [17, 18]. Our findings highlight a bad or neither good nor bad lockdown experience in almost half of patients. These findings confirm that social distancing during lockdown was associated with less satisfied persons [1, 5, 19]. Patients with chronic pain are more likely to suffer from self-isolating and it can be assumed a mediating role on perceived changes in pain during lockdown and its experience [8]. Moreover, amplification of bodily sensations’ perception or change as symptoms of being ill which impacts on chronic pain experience was recently shown to be exacerbated by the current pandemic, particularly in vulnerable populations [8, 20]. Finally, bad experience is usually associated with changes in health behavior, in particular tobacco consumption and alcohol misuse [4, 21]. However, we did not observe a clear increase in psychoactive substances consumptions intended to reduce pain in our sample.

COVID-19 lockdown has caused significant changes in individuals’ lifestyle and has been likely to decrease the number of times per week devoted to participating PA and to increase the time spent sitting. This negative effect seems to affect a large proportion of people around the world and all PA intensity levels (vigorous, moderate, walking, and overall), and an increase from 16 to 40% of people spending more than 8 h a day in sedentary activities could even be observed [4, 6]. The reported overall decline in PA is likely a consequence of social distancing, travel restrictions, closure of usual exercise venues, etc. Moreover, recent study reported a lower LBP prevalence among individuals who regularly practiced PA and the negative effect of prolonged sitting on LBP intensity [22–24]. The results of our study concurred with these findings. Indeed, perceived decrease in levels of PA seems to be independently related to perceptions of increased pain and physical inactivity promotes physical deconditioning which exacerbates LBP during activity [8, 25]. Previous studies have also highlighted the importance of catastrophizing in chronic pain population during response to high-stress situations [26, 27]. In chronic LBP patients, catastrophizing contributes to hypervigilance and fear related to pain and results in avoidance of daily activities and decrease levels of PA [28] but we, unfortunately, did not measure this parameter.

For most employees, teleworking which has been implemented by many companies during lockdown has been the first experience. Although teleworking allows reducing commuting time, and better control overtime schedule, it has the disadvantages of monitoring performance, communication problems, no clear separation between home and work tasks, and unsuitability with all works [29]. Moreover, the home environment is likely to be faulty in many aspects. In particular, the absence of ergonomic office facilities at home may impede the adoption of a healthy posture and may promote the onset of musculoskeletal disorders [25, 30, 31]. Homeworking may cause also stress, and isolation, which influences job effectiveness, well-being, and work-life balance [30]. Our results showed that teleworking seemed to affect LBP significantly. Firstly, effect of teleworking on LBP could have been underestimated in our study because subjects were practicing this job type for a too-brief period to produce the adverse effects [31]. Secondly, the multifactorial nature of LBP, and in particular, psychosocial factors (stress, job satisfaction, isolation, etc.) could have played a role in LBP course and that could be the reason why teleworking did not appear as a separate associated risk in our multivariate analysis. Finally, the French Public Health Institute found that the incidence of LBP was about 2.5 times higher among workers who had been newly placed on telework during lockdown and that among workers with pre-existing LBP, those who had continued telework, as usual, had more luck to see their LBP evolve favorably, but our study did not allow us to examine these two types of situations [32].

## Strengths and limitations

The strength of this study is that the data were collected very quickly after the first COVID-19 lockdown using a fully anonymous questionnaire. The multicenter design of the study allowed the inter-regional variability of the lockdown conditions to be taken into account. Regarding the methodological issues, possible limitations could be related to (1) the cross-sectional design assessing “before” lockdown data retrospectively, (2) the self-administered and anonymous computerized questionnaire which prevented us from establishing an accurate flowchart of respondents. However, given that the lockdown was a sudden measure, we were obviously not able to develop and spread the survey “before” lockdown to have an ideal control condition, and (3) the study was carried out among patients from tertiary centers of chronic LBP management. To our knowledge, no study has specifically looked at the consequences of confinement in chronic LBP patients specifically. Our results highlight the deleterious impact of confinement on LBP and improve our understanding of the involved factors.

## Conclusion

The lockdown due to the COVID-19 pandemic led to a tendency for stable to worsening LBP intensity among chronic LBP patients. Our results reveal psycho-social pressure during lockdown. Moreover, chronic LBP patients are already at an increased risk of being hypoactive which was aggraded by lockdown. Given the established benefits of PA on LBP, additional strategies to promote PA are needed. Lockdown encouraged greater use of Information and Communications Technology (ICT). Therefore, future PA and/or psychological support interventions to foster a more active and healthy lockdown lifestyle during pandemic could be based on ICT solutions. Finally, teleworking exposes particularly workers to ergonomic risk factors likely to increase LBP intensity. Particular intention should be paid to the workplace comfort at home.

## Supplementary Information

Below is the link to the electronic supplementary material.

Supplementary file1 (DOCX 75 kb)
