# Supplementary material for: Effects of COVID-19 lockdown on low back pain intensity in chronic low back pain patients: results of the multicenter CONFI-LOMB study
Source: Eur Spine J. 2021 Oct 4;31(1):159–66. doi: 10.1007/s00586-021-07007-8 (PMC8488322; doi:10.1007/s00586-021-07007-8)
Supplement: Supplementary file 1 — Supplementary file1 (DOCX 75 kb) [file 586_2021_7007_MOESM1_ESM.docx]

***Questionnaire CONFI-LOMB***

**Dossier n°** ⏐__⏐__⏐ -⏐__⏐__⏐__⏐

n° centre n° patient

***Vous concernant***

1. **Vous êtes :**  Un homme  Une femme
2. **Merci d’indiquer votre âge :** __________ans
3. **Vous êtes :** Seul En couple
4. **Merci d’indiquer le numéro du département de votre lieu de résidence habituel :** __________
5. **Avant la période de confinement, vous étiez :**

En emploi (quel que soit le type de contrat de travail)  Sans profession (femme au foyer, etc.)

Au chômage ou en recherche d’emploi  Étudiant(e)  Retraité(e)

Autre *(merci de préciser)* : ________________________________

1. **Si vous êtes en emploi, merci d’indiquer votre catégorie professionnelle :**

Agriculteurs

Artisans, commerçants et chefs d'entreprises

Cadres et professions intellectuelles supérieures

Professions intermédiaires (agent de maîtrise, technicien supérieur.......)

Employés

Ouvriers

Autre *(merci de préciser)*:___________________________________

***Concernant le confinement***

1. **Au cours du confinement, vous étiez confiné(e) :**  Seul(e)  Avec d’autres personnes
2. **Etiez-vous confiné(e) avec** :  Un ou plusieurs enfant(s) en bas âge

Une ou plusieurs personne(s) âgée(s) ou dépendante(s)

1. **Sur votre lieu de confinement, aviez-vous l’impression d’avoir suffisamment d’espace pour vous-même ?**

Oui  Non

1. **Comment vivez-vous cette période de confinement ?**

Très bien  Plutôt bien Ni bien ni mal ⁪  Plutôt mal Très mal

***Concernant le coronavirus***

1. **Avez-vous été atteint(e) par le Coronavirus personnellement ?** Oui  Non
2. **Si oui, le diagnostic a-t-il été confirmé ?** *(par un médecin, un test ou un scanner)*  Oui  Non
3. **Si oui, quelle était la gravité de l’infection ?**

Peu ou pas de symptômes

Symptômes limitant fortement vos activités quotidiennes (forte fièvre, essoufflement, grande fatigue, etc.)

Vous avez été hospitalisé(e) dans un service traditionnel

Vous avez été hospitalisé(e) dans un service de réanimation

***Concernant votre problème de dos***

1. **Merci d’indiquer votre taille :** ________m______
2. **Merci d’indiquer votre poids de forme avant le confinement :** ______________ Kg
3. **Merci d’indiquer votre poids actuel**______________ Kg
4. **Depuis combien de temps avez-vous des problèmes de dos ?**

Moins de 3 mois  3 à 6 mois  6 mois à 2 ans plus de 2 ans

1. **Selon vous, pendant le confinement, votre douleur du dos :**

S’est beaucoup améliorée              s’est modérément améliorée

S’est légèrement améliorée  Est restée stable  S’est légèrement aggravée

S’est modérément aggravée  S’est beaucoup aggravée​

Pour les deux questions suivantes, nous aimerions que vous indiquiez l’intensité de votre douleur en faisant une croix sur une ligne allant de 0 à 10 (0 représente aucune douleur et 10 la pire douleur imaginable) ; Voici un exemple :

| **0 1 2 3 4 5 6 7 8 9 10**  **I-----------------------------------------------------------------I**  **Aucune douleur La pire douleur imaginable** |
| --- |

1. **Merci d’indiquer l’intensité de votre douleur du dos durant les semaines qui ont précédé le confinement** *(fin février / début mars 2020)*

| **0 1 2 3 4 5 6 7 8 9 10**  **I-----------------------------------------------------------------I**  **Aucune douleur La pire douleur imaginable** |
| --- |

1. **Merci d’indiquer l’intensité de votre douleur de dos durant la dernière semaine du confinement (semaine du 4 au 10 mai)**

| **0 1 2 3 4 5 6 7 8 9 10**  **I-----------------------------------------------------------------I**  **Aucune douleur La pire douleur imaginable** |
| --- |

1. **Pendant le confinement, avez-vous du augmenter votre traitement et/ou consulter un médecin pour une aggravation de vos douleurs du dos ?**  Oui  Non
2. **Pendant le confinement, avez-vous pu diminuer votre traitement du fait d’une diminution de vos douleurs du dos ?**  Oui  Non
3. **Pendant le confinement, vos douleurs du dos vous ont-elles incité(e) à augmenter votre consommation de :**

**Tabac**  Oui  Non

**Alcool**  Oui  Non

**Cannabis**  Oui  Non

**Traitements contre l’anxiété / tranquillisants**  Oui  Non

***Concernant vos activités physiques et sportives***

1. **Merci de cocher les cases correspondant le mieux à votre pratique d’activités physiques et sportives avant et pendant le confinement :**

|  | ***Avant***  le confinement | ***Pendant***  le confinement |
| --- | --- | --- |
| Jamais ou rarement |  |  |
| Moins de 2 heures / semaine |  |  |
| 2 à 4 heures / semaine |  |  |
| 4 à 6 heures / semaine |  |  |
| Plus de 6 heures / semaine |  |  |

1. **Pendant le confinement, avez-vous utilisé la possibilité de déplacement dérogatoire pour pratiquer une activité physique ou sportive telle que la course à pied, la marche à pied ?**

Jamais ou rarement  Moins de 2 heures / semaine  2 à 4 heures / semaine

4 à 6 heures / semaine  Plus de 6 heures / semaine

1. **Merci de cocher les cases correspondant le mieux à vos activités sédentaires** (c’est-à-dire le temps passé sur les écrans (TV, smartphone, tablette, etc.), à la lecture, à travailler au bureau, etc.) **avant et pendant le confinement**:

|  | ***Avant***  le confinement | ***Pendant***  le confinement |
| --- | --- | --- |
| Moins de 3 heures / jour |  |  |
| 3 à 7 heures / jour |  |  |
| 7 à 10 heures / jour |  |  |
| Plus de 10 heures / jour |  |  |

***Si vous étiez en activité professionnelle avant et/ou pendant le confinement, merci de répondre aux questions suivantes :***

1. **Pendant le confinement, avez-vous continué à travailler ?**  Oui  Non
2. **Si oui, votre temps de travail était-il ?**

Réduit  Identique  Augmenté

1. **Pendant le confinement, avez-vous fait au moins en partie du télétravail ?**  Oui  Non
2. **Si oui, pensez-vous que le télétravail, pendant le confinement :**

A aggravé votre mal de dos

N’a pas eu d’influence sur votre mal de dos

A amélioré votre mal de dos

1. **Si vous faisiez et/ou faites encore du télétravail, disposez-vous d’un poste de travail** *(bureau ou équivalent)* **dédié à votre activité de télétravail ?**

Oui  Non

1. **Si vous faisiez et/ou faites encore du télétravail, votre matériel** *(ordinateur, modem, télécopieur, webcam)* **et votre installation** *(bureau, siège)* **sont-ils adaptés ?**

Oui  Non
